# Rhizobial Volatiles: Potential New Players in the Complex Interkingdom Signaling With Legumes

**DOI:** 10.3389/fpls.2021.698912

**Published:** 2021-06-22

**Authors:** María J. Soto, Isabel M. López-Lara, Otto Geiger, María C. Romero-Puertas, Pieter van Dillewijn

**Affiliations:** ^1^Estación Experimental del Zaidín, CSIC, Granada, Spain; ^2^Centro de Ciencias Genómicas, Universidad Nacional Autónoma de México, Cuernavaca, Mexico

**Keywords:** *Rhizobium*, volatile compounds, signaling, symbiosis, plant defense, iron uptake, interkingdom communication

## Abstract

Bacteria release a wide range of volatile compounds that play important roles in intermicrobial and interkingdom communication. Volatile metabolites emitted by rhizobacteria can promote plant growth and increase plant resistance to both biotic and abiotic stresses. Rhizobia establish beneficial nitrogen-fixing symbiosis with legume plants in a process starting with a chemical dialog in the rhizosphere involving various diffusible compounds. Despite being one of the most studied plant-interacting microorganisms, very little is known about volatile compounds produced by rhizobia and their biological/ecological role. Evidence indicates that plants can perceive and respond to volatiles emitted by rhizobia. In this perspective, we present recent data that open the possibility that rhizobial volatile compounds have a role in symbiotic interactions with legumes and discuss future directions that could shed light onto this area of investigation.

## Introduction

Microorganisms can produce a broad variety of chemical signals, many of which play important roles in communication with neighboring organisms. In addition to the better-studied small diffusible chemical signals, in the last decades microbial volatile compounds (VCs) are receiving increased attention. Accumulating evidence indicates that VCs are not just by-products of microbial metabolism but also exhibit relevant biological activities with important roles in intermicrobial communication and interkingdom interactions with eukaryotic hosts (Weisskopf et al., 2021). Microbial volatiles are characterized by their low molecular mass (<300 Daltons) and high vapor pressure, properties that facilitate their dispersal in air and water over long distances (Schulz and Dickschat 2007). Volatiles emitted by bacteria exhibit diverse chemical structures, comprising inorganic (CO_2_, CO, NO, H_2_S, NH_3_, or HCN) and organic compounds, with the latter belonging to various chemical classes, such as terpenes, aromatic compounds, hydrocarbons, ketones, alcohols, aldehydes, acids, or nitrogen- and sulfur-containing metabolites (Schulz and Dickschat 2007; Audrain et al., 2015; Lemfack et al., 2018). The amount and profile of species-specific and generic volatiles produced by a microorganism (i.e., volatilome) vary in response to numerous factors including the availability of nutrients and oxygen, humidity, pH, temperature, growth phase, and even the presence of other organisms (Schmidt et al., 2015, 2017; Misztal et al., 2018).

Bacterial volatile metabolites can influence important physiological processes in numerous bacteria, fungi, and plants. Some of them negatively influence the growth and virulence of microorganisms, characteristics that could be harnessed in the design of new strategies to fight against the rise of antibiotic resistance in pathogens (Avalos et al., 2018). Bacterial volatiles are especially well known for their capacity to increase plant growth and resistance against both biotic and abiotic stresses. These properties could be exploited for the development of eco-friendly solutions in the form of biofertilizers and biopesticides to improve plant health and productivity (Kanchiswamy et al., 2015; Chung et al., 2016; Sharifi and Ryu, 2018; Garbeva and Weisskopf, 2020; Thomas et al., 2020).

Considering the relevant effects of bacterial volatiles in intermicrobial and interkingdom communication, it is surprising that very little is known about the production and ecological role of volatile compounds emitted by soil bacteria that establish nitrogen-fixing symbiosis with legumes, collectively referred to as rhizobia. Paradoxically, rhizobia are among the best-known plant-interacting microorganisms and they have frequently been used as inoculants to improve N fertilization of legume plants. Perhaps, efforts focused on the nodulation and nitrogen fixation processes, which are the most relevant attributes of these plant endosymbionts, have contributed that some other properties of these rhizobacteria have been overlooked. In this perspective, we present recent data that open the possibility that rhizobial volatiles have a role in the interkingdom communication with plants that could impact the outcome of symbiotic interactions with legumes as well as other plant-microbe interactions.

## Overview of the Role of Bacterial Volatile Compounds in Intermicrobial and Interkingdom Interactions

The effects of bacterial volatile compounds (BVCs) in microbe-microbe interactions are diverse and have been described in several reviews (Audrain et al., 2015; Schmidt et al., 2015; Schulz-Bohm et al., 2017; Tyc et al., 2017; Weisskopf et al., 2021). These bacterial airborne metabolites can either positively or negatively influence the physiology of different microorganisms. Some have strong antimicrobial effects, inhibiting the growth of fungi and/or bacteria. Others act as infochemical molecules that, at a distance, are capable of altering gene expression and important behaviors, such as motility, biofilm formation, virulence, development, or stress and antibiotic resistance. The same volatile can elicit different or even opposing responses depending on the interacting organism, highlighting the importance of deciphering the mechanism of action of these compounds. Up to now, very little is known about how microorganisms perceive and respond to the wide spectrum of BVCs, and this represents an intense research area. Alteration of membrane permeability, induction of pH changes in the medium, oxidative stress mitigation, or interference with quorum sensing regulation are some of the mechanisms that have been shown to play a role in the microbial responses to different volatiles (reviewed in Weisskopf et al., 2021).

While the role of BVCs in intermicrobial interactions has only recently been acknowledged, their functions in interkingdom interactions with plants have been known for almost 20 years (reviewed in Sharifi and Ryu, 2018; Garbeva and Weisskopf, 2020; Thomas et al., 2020; Weisskopf et al., 2021). Since the seminal contribution by Ryu et al. (2003) reporting that volatile blends emitted by two *Bacillus* species were able to promote growth of *Arabidopsis*, numerous studies have described the different and relevant effects of BVCs on plants. Plant growth promotion is a common property of volatile mixtures emitted by rhizosphere bacteria (Blom et al., 2011). However, the molecular bases responsible for this effect are still poorly understood. Stimulation of photosynthesis, root growth, and the uptake of specific nutrients, such as iron and sulfur, are some of the mechanisms known to contribute to the BVC-mediated plant growth promotion (Ryu et al., 2003; Zhang et al., 2008, 2009; Meldau et al., 2013). Interestingly, BVCs can also help plants to cope with abiotic and biotic stresses. BVC-mediated plant tolerance to abiotic stresses (drought, salinization, and heavy metal toxicity) is achieved by increasing osmoprotectants, antioxidant activities, Na^+^ homeostasis, or sulfur-containing metabolites (reviewed in Sharifi and Ryu, 2018). An important property of some BVCs is their ability to protect plants from pathogenic microorganisms, an effect that could be the result of both direct inhibition of pathogen growth/virulence and the activation of plant immunity (i.e., induced systemic resistance), although the latter seems to play a more important role (Bailly and Weisskopf, 2017). Interestingly, rhizobacteria produce volatiles that can facilitate mutualistic associations between plants and beneficial bacteria without compromising resistance against phytopathogens in a process dependent on phosphorus availability for the plant (Morcillo et al., 2020).

In the different plant responses triggered by BVCs, signaling molecules, such as reactive oxygen species and NO and the modulation of the biosynthesis, perception, and homeostasis of different phytohormones, play a pivotal role (Sharifi and Ryu, 2018; Tyagi et al., 2018). Ethylene, auxin, cytokinin, abscisic acid, and gibberellin are involved in BVC-mediated plant growth promotion, while ethylene, jasmonic acid, and salicylic acid are crucial for BVC-induced plant defense responses.

## *Rhizobium*-Legume Symbosis: Complex Signal Exchange in the Rhizosphere

**Figure 1:**
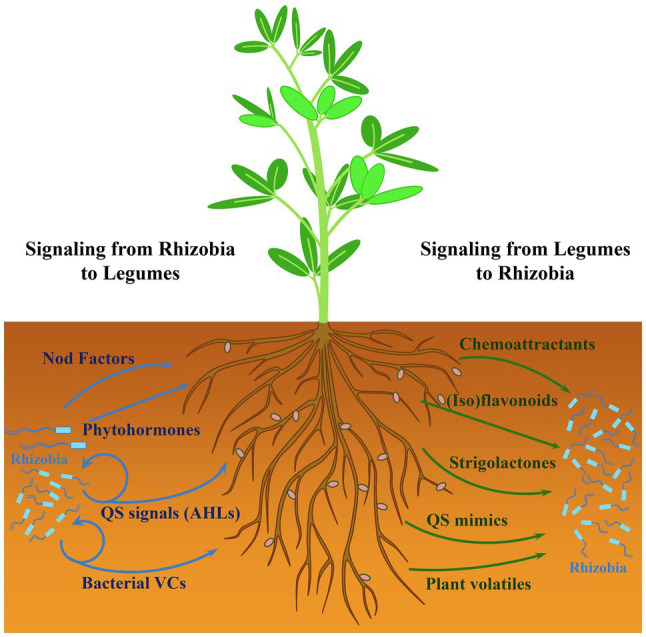
Interkingdom signaling in the rhizosphere between rhizobia and legumes. The effects of bacteria- and plant-derived signals are shown with blue and green arrows, respectively. Bacterial quorum sensing (QS) signals (i.e., *N*-acyl-homoserine lactones, AHLs) and volatile compounds (VCs) also play a role in intermicrobial communication.

The development of nitrogen-fixing root nodules characteristic of the *Rhizobium*-legume symbiosis is the outcome of a process that involves a complex signal exchange between the host plant and rhizobia (Oldroyd, 2013; Poole et al., 2018; Roy et al., 2020). This interkingdom signaling initiates in the rhizosphere, i.e., the region of soil under the influence of plant roots (Bakker et al., 2013). In this niche, several plant- and bacteria-derived diffusible compounds have been shown to participate in the early dialog established between the two organisms (Figure 1). Some of the signaling compounds are crucial for root nodulation. This is the case of plant root-exuded (iso)flavonoids, which induce bacterial *nod* genes, leading to the synthesis and secretion of lipochitooligosaccharides, also known as Nod factors. This key bacterial signal is perceived by the plant *via* specific receptors to activate the symbiosis signaling pathway required for rhizobial infection and nodule formation while, at the same time, overriding host defense reactions triggered during rhizobial invasion (Zipfel and Oldroyd, 2017; Roy et al., 2020). Besides the well-known (iso)flavonoids and Nod factors, additional legume- and rhizobia-produced diffusible molecules described below participate in an interkingdom communication that is not essential for nodule organogenesis but contribute to fine-tune some aspects of the symbiosis.

Compounds present in legume root exudates, such as amino acids, quaternary ammonium compounds, and organic acids function as chemoattractants for rhizobia. Upon perception by specific bacterial chemoreceptors, they promote bacterial movement toward plant roots facilitating root colonization and the possibility of finding proper sites for infection (Scharf et al., 2016; Compton et al., 2020; Compton and Scharf, 2021). Root exudates can also contain various low molecular weight compounds that mimic bacterial signals and affect quorum sensing (QS) regulation in rhizobia, thereby enhancing or inhibiting the phenotypes controlled by this cell density-dependent regulatory mechanism (Gao et al., 2003; Mathesius et al., 2003; Bauer and Mathesius, 2004). In rhizobia, QS usually relies on the production and perception of *N*-acyl-homoserine lactones (AHLs) that control important functions, some of which are relevant for the interaction with the legume host (Calatrava-Morales et al., 2018). Strigolactones (SLs), a group of carotenoid-derived plant hormones exuded by roots, participate in the interaction of plants with beneficial soil microorganisms (López-Ráez et al., 2017). SLs have a positive influence on nodulation by promoting infection thread formation, in a process in which they may act as a signal for the bacterial partner (Soto et al., 2010; McAdam et al., 2017). In fact, a synthetic SL promotes bacterial surface motility in the alfalfa symbiont *Sinorhizobium* (*Ensifer*) *meliloti* (Peláez-Vico et al., 2016). Plant roots also produce a plethora of volatile compounds that participate in different trophic interactions belowground, including the attraction of beneficial bacteria (Schulz-Bohm et al., 2018). The role of these plant-derived chemical signals in communication with rhizobia deserves attention but is not discussed in this perspective.

Besides Nod factors, phytohormones and AHLs produced by rhizobia are the main diffusible signals participating in the interkingdom signaling with legumes to impact the establishment of symbiosis (Figure 1). The ability to synthesize all major phytohormones (auxin, cytokinin, abscisic acid, and gibberellins) has been described among rhizobial species (Ferguson and Mathesius, 2014). The production of these signals by the microbial partner affects the establishment of the symbiosis by controlling nodulation and nitrogen fixation (Ferguson and Mathesius, 2014). AHL-type QS signals for intra- and interspecies communication are also perceived by legumes, leading to different responses, such as changes in root protein content and secretion of signal-mimic compounds, that can influence the outcome of the symbiosis (Mathesius et al., 2003; Veliz-Vallejos et al., 2014, 2020). Although less well studied than in other rhizobacteria, volatile compounds (VCs) are also produced by symbiotic rhizobia. Recent reports have shown that these rhizobial metabolites can trigger plant responses and interfere with the establishment of plant-bacteria interactions (Orozco-Mosqueda et al., 2013; Sánchez-López et al., 2016; Hernández-Calderón et al., 2018; López-Lara et al., 2018). These findings support the notion that rhizobial volatiles might be additional players in the interkingdom signaling with legumes and whose precise role in symbiosis requires further investigation.

## Bioactivities Assigned to Rhizobial Volatiles

The production of VCs by rhizobia and its effects on plants have been little investigated. One of the first studies analyzed the VCs produced by *S. meliloti* in the absence or the presence of volatiles emitted by *Medicago truncatula* seedlings (Orozco-Mosqueda et al., 2013). Some of the *S. meliloti* VCs detected like 2-methyl-1-propanol and dimethyl-disulfide were previously described in the volatilomes of different rhizobacteria. Particularly noteworthy was the identification of five compounds that were detected only when the plant and bacteria were co-cultivated in the same Petri dish without physical contact, thereby suggesting the existence of an interkingdom communication whereby the plant, the bacterium, or both were able to detect and respond to the volatiles produced by the other interacting organism.

Interestingly, two studies have shown that VCs emitted by two different rhizobial species, *S. meliloti* and *Sinorhizobium fredii*, were capable of promoting growth of the non-legume plants *Sorghum bicolor* and *Arabidopsis* (Sánchez-López et al., 2016; Hernández-Calderón et al., 2018). Moreover, exposure to VCs emitted by *S. meliloti* activated iron-uptake mechanisms, namely rhizosphere acidification and increased root ferric reductase, in *M. truncatula* (Orozco-Mosqueda et al., 2013), and increased chlorophyll content and transcriptional activity of iron-uptake genes in *S. bicolor* (Hernández-Calderón et al., 2018). These observations led the authors to suggest that the rhizobial VC-mediated phytostimulatory effect could be caused by improving the plants’ iron content, as has been shown for other beneficial rhizobacteria (Zhang et al., 2009). However, this possibility still needs to be experimentally tested. This is an important issue because accumulating evidence indicates that the plant iron-deficiency response is linked to the activation of defense responses (Koen et al., 2014; Romera et al., 2019). Indeed, volatile blends emitted by beneficial rhizobacteria are able to trigger both plant defense and Fe deficiency responses (Zamioudis et al., 2015). In agreement with this, exposure of *S. bicolor* to *S. meliloti* VCs not only induced the expression of Fe-uptake genes but also that of plant-defense genes (Hernández-Calderón et al., 2018). The coordinated activation of the iron-deficiency and defense responses in plants has also been shown in legumes exposed to pure volatiles. Medium supplemented with *N,N*-dimethylhexadecylamine (DMHDA), a volatile produced during the co-cultivation of *S. meliloti* and *M. truncatula* (Orozco-Mosqueda et al., 2013), has been shown to promote plant growth and induce iron-deficiency and defense response genes in *M. truncatula* (Montejano-Ramírez et al., 2020). However, effects of either DMHDA or volatile blends of rhizobia in the establishment of the *Rhizobium*-legume symbiosis have not been reported yet.

To the best of our knowledge, only one study associates the effect of a rhizobial VC with the *Rhizobium*-legume symbiosis. The methylketone 2-tridecanone (2-TDC) was identified as the volatile responsible for the pleiotropic phenotype shown by a *S. meliloti* mutant impaired in alfalfa root colonization and exhibiting increased surface motility and defects in biofilm formation (López-Lara et al., 2018). 2-TDC is known as a natural insecticide produced by wild varieties of tomato plants (Williams et al., 1980), and its production was reported in several bacterial species, including different rhizobacteria (Blom et al., 2011; Lemfack et al., 2018). The results described by López-Lara et al. (2018) indicate that 2-TDC is an infochemical able to affect important bacterial traits, such as surface motility and biofilm formation. Moreover, 2-TDC negatively interfered with different plant–bacteria associations, hampering alfalfa nodulation by *S. meliloti* and also the development of tomato bacterial speck disease by *Pseudomonas syringae* pv *tomato* (López-Lara et al., 2018). It was also shown that 2-TDC hinders the bacterial ability to efficiently colonize plant tissues. However, it remains unknown whether this is the result of 2-TDC altering bacterial behaviors required for plant colonization, and/or that the ketone elicits plant responses that have a negative effect on the interacting bacteria.

## Future Perspectives

**Figure 2:**
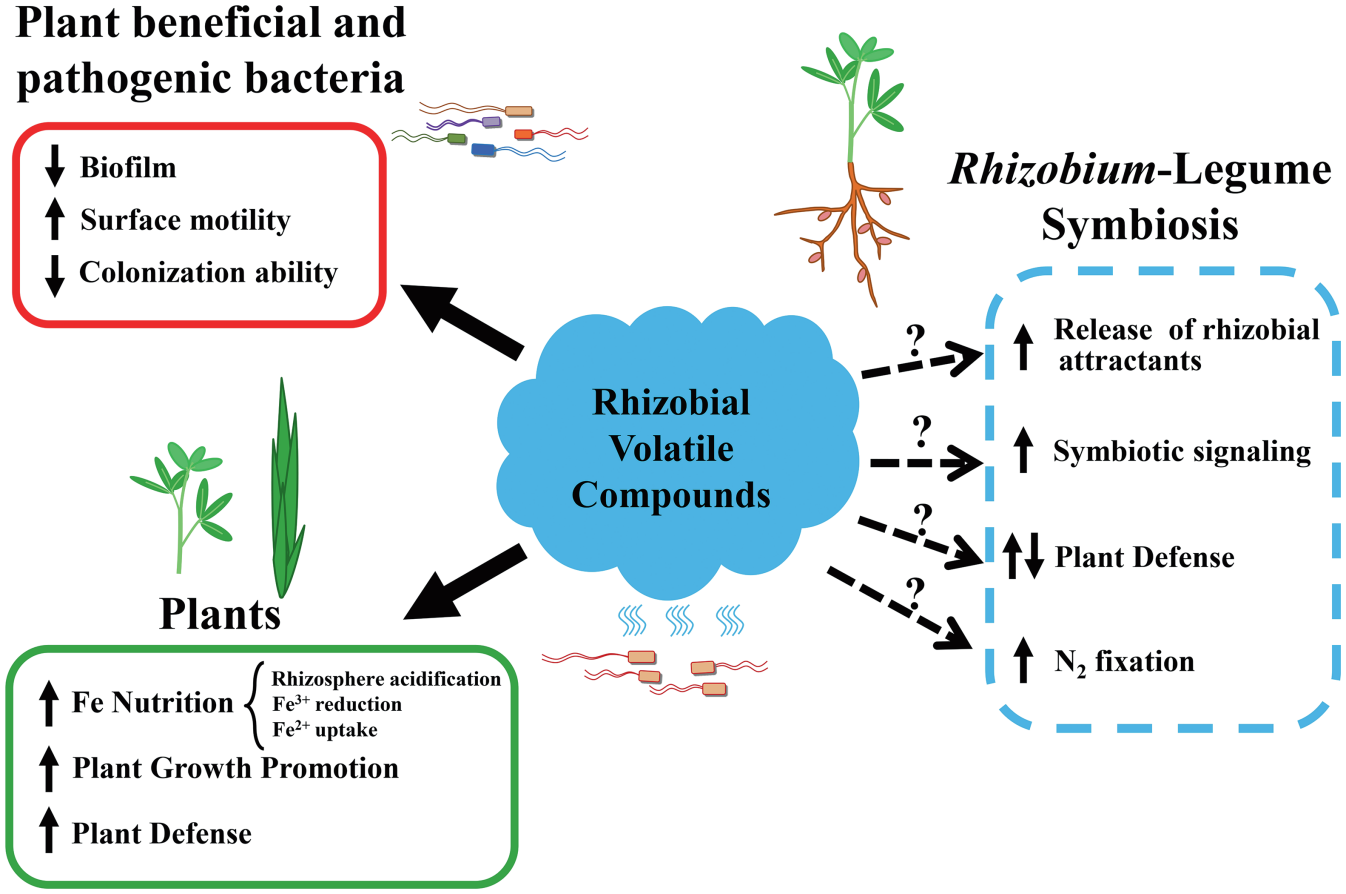
Recognized and putative biological activities of volatile compounds emitted by rhizobia. Bioactivities associated with rhizobial volatile compounds in the literature are shown on the left side of the figure and indicated with solid arrows. Effects on plant beneficial and pathogenic bacteria caused by the discrete application of a rhizobial volatile are shown in a red box. Effects on legume and non-legume plants triggered by rhizobial volatilomes are shown in a green box. Putative effects of rhizobial volatiles, as either volatile blends or discrete compounds, with an impact on the *Rhizobium*-legume symbiosis are shown on the right side of the figure and indicated with dashed arrows and question marks as hypotheses that need to be evaluated.

Evidence clearly indicates that plants can perceive and respond to volatile blends emitted by rhizobia. Plant growth promotion, activation of iron-uptake mechanisms, and increased transcription of defense genes are some of the responses detected in plants exposed to rhizobial VCs. However, many questions remain concerning the effects of rhizobial VCs on their host plants, and specifically on the establishment of efficient symbiosis (Figure 2).

The ability of bacterial volatiles to trigger plant defense responses led to the suggestion that these airborne metabolites could be considered as Microbe-Associated Molecular Patterns (MAMPs; Sharifi and Ryu, 2018). MAMPs are usually conserved microbial molecules, which are perceived by plant cell surface transmembrane receptors and activate a basal defense known as the MAMP-triggered immunity (Jones and Dangl, 2006). Up to now, rhizobial VC-mediated activation of plant defense responses has only been reported in a non-host monocotyledonous plant (Hernández-Calderón et al., 2018). Since the development of nitrogen-fixing nodules requires the strict and continuous control of plant immunity (Cao et al., 2017; Berrabah et al., 2019; Benezech et al., 2020), evaluation of defense responses in legumes exposed to rhizobial VCs deserves special attention and could give clues about the contribution of these metabolites to the *Rhizobium*-legume symbiosis. Moreover, the finding that a rhizobial produced volatile (2-TDC) can protect plants against bacterial phytopathogens through an as yet unknown mechanism (López-Lara et al., 2018) opens the possibility of using rhizobia and their volatilomes as new biocontrol solutions and sources for new biopesticides.

Interestingly, bacterial volatiles can also facilitate mutualistic associations with beneficial rhizobacteria without compromising disease resistance (Morcillo et al., 2020). Whether rhizobial VCs can also facilitate symbiosis with their legume hosts by triggering plant-specific responses to attract the microsymbiont and/or by activating the symbiosis signaling pathway are aspects worth investigating.

The activation of root iron-uptake mechanisms has been detected in both legumes and non-legumes exposed to rhizobial VCs. This effect could be linked to the activation of plant defense responses as has been shown for VCs of other beneficial rhizobacteria (Zamioudis et al., 2015; Romera et al., 2019). The molecular bases responsible for the coordinated regulation of the plant iron-deficiency response and plant immunity have been investigated mainly in *Arabidopsis* where several phytohormones, signaling molecules as well as a transcription factor have been shown to be involved in regulating both processes (Romera et al., 2019). Whether similar regulatory mechanisms participate in rhizobial VC-mediated effects in legumes awaits further investigation. Moreover, considering that symbiotic nitrogen fixation is a Fe-demanding process, the VC-mediated activation of iron-uptake mechanisms in legume roots could contribute to the efficiency of the symbiosis.

Nitrogen availability in the soil is the major factor determining whether the plant will establish nitrogen-fixing symbiosis with rhizobia (Streeter and Wong, 1988). The molecular bases underlying this regulatory mechanism are starting to be deciphered (Chaulagain and Frugoli, 2021). Recently, it has been shown that the availability of a nutrient, namely phosphate, determines the plant’s response to a bacterial volatile to result in either mutualism or increased defense responses (Morcillo et al., 2020). Therefore, to assess whether soil nitrogen conditions modulate the legume response to rhizobial VC is another interesting area of research.

Microbial VCs are considered early signaling molecules whose effects in plants depend on the compound’s concentration and the plant developmental stage, but the plant receptors and regulatory pathways involved in their recognition are still largely unknown (Weisskopf et al., 2021). The biological activities associated with rhizobial VCs suggest that they could be additional players in the early signaling with legumes and impact the establishment of symbiosis. Further investigation of legume responses to rhizobial VCs, both as volatile blends and as discrete compounds, and ideally using experimental setups that better simulate complex rhizosphere conditions (Kai et al., 2016) will help to elucidate their specific roles in symbiosis and shed light on how plants perceive these signals.

## Data Availability Statement

The original contributions presented in the study are included in the article/supplementary material, and further inquiries can be directed to the corresponding author.

## Author Contributions

MS conceived and wrote most of the sections. PD made all the drawings. PD, IL-L, OG, and MR-P contributed to the writing and editing of the manuscript. All authors read and approved the manuscript.

### Conflict of Interest

The authors declare that the research was conducted in the absence of any commercial or financial relationships that could be construed as a potential conflict of interest.
